# The prognostic significance and value of cyclin D1, CDK4 and p16 in human breast cancer

**DOI:** 10.1186/bcr3376

**Published:** 2013-01-21

**Authors:** Emmi Peurala, Peppi Koivunen, Kirsi-Maria Haapasaari, Risto Bloigu, Arja Jukkola-Vuorinen

**Affiliations:** 1 Department of Oncology and Radiotherapy, Oulu University Hospital, and University of Oulu, Kajaanintie 50, P.O. Box 22, 90029 Oulu, Finland; 2 Department of Medical Biochemistry and Molecular Biology and Oulu Center for Cell-Matrix Research, University of Oulu, Aapistie 7, P.O. Box 5000, 90014 Oulu, Finland; 3 Department of Pathology, University of Oulu, Aapistie 5b, P.O. Box 5000, 90014 Oulu, Finland; 4 Medical Informatics and Statistics Research Group, University of Oulu, Aapistie 7, P.O. Box 5000, 90014 Oulu, Finland

## Abstract

**Introduction:**

Loss of the retinoblastoma protein tumor suppressor gene (*RB*) coding for a nuclear phosphoprotein that regulates the cell cycle is found in many human cancers and probably leads to disruption of the p16-cyclin D1-CDK4/6-RB pathway. Cyclin D1 is known to activate CDK4, which then phosphorylates the RB protein, leading to cell cycle progression. p16 inhibits CDK4, keeping RB hypophosphorylated and preventing cell cycle progression. The significance of these three markers, cyclin D1, CDK4 and p16, for breast cancer and carcinogenesis is nevertheless still controversial.

**Methods:**

The material consisted of 102 formalin-fixed human breast cancer samples, in which cyclin D1, CDK4 and p16 expression was evaluated immunohistochemically. The amounts of cyclin D1 mRNA present were analyzed by quantitative real time PCR.

**Results:**

High cyclin D1 expression statistically significantly correlated with lower tumor grade, estrogen and progesterone receptor positivity and lower proliferation activity in breast tumors and increased breast cancer-specific survival and overall survival. Tumors with high cyclin D1 protein had 1.8 times higher expression of cyclin D1 mRNA. CDK4 expression did not correlate with cyclin D1 expression or the survival data. p16 expression was associated with Human Epidermal Growth Factor Receptor 2 (HER2) negativity and increased breast cancer-specific survival and disease-free survival. No statistical correlations between cyclin D1, CDK4 and p16 were found.

**Conclusions:**

Cyclin D1 was associated with a good breast cancer prognosis but functioned independently of CDK4. High cyclin D1 expression may be partially due to increased *CCND1 *transcription. p16 correlated with a better prognosis and may function without CDK4. In conclusion, it appears that cyclin D1, CDK4 and p16 function independently in human breast cancer.

## Introduction

**Figure 1:**
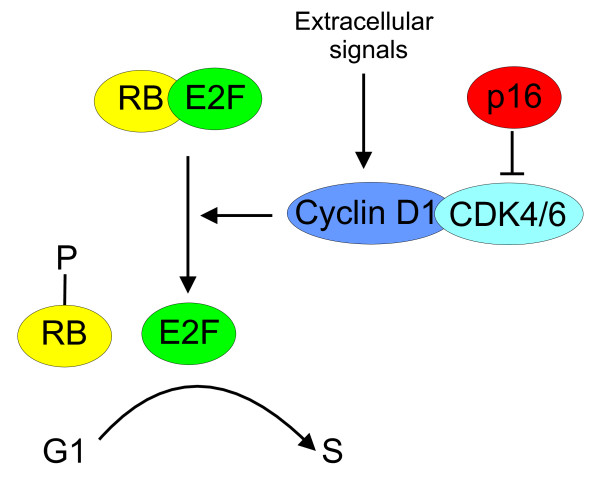
**Regulation of the cell cycle G1/S transition by cyclin D1, CDK4 and p16**. RB, retinoblastoma protein, CDK4/6, cyclin-dependent kinase 4/6.

The retinoblastoma tumor suppressor gene (*RB*) encodes a nuclear phosphoprotein that plays a central role in regulating the cell cycle [1]. RB regulates progression through the G1-to-S phase transition of the cell cycle [1]. Loss of *RB *is well documented in many human tumor types and it is probable that the p16-cyclin D1-CDK4/6-RB pathway is disrupted in most human malignancies [2]. Extracellular signals induce the expression of cyclin D1 in cells entering the cell cycle and this binds to and activates cyclin-dependent kinases (CDK4 and CDK6) (Figure 1) [1-5]. The ensuing complexes in turn lead to the phosphorylation of RB, resulting in its dissociation from the transcription factors, predominantly members of the E2F family, which then activate the many genes required for progression of the cell cycle to the S phase (Figure 1) [1-3]. p16, also known as p16^INK4a^, a member of the INK4 family of CDK inhibitors, inhibits CDK4 and CDK6, maintaining RB in its hypophosphorylated E2F-associated state, and thereby preventing G1-to-S phase progression (Figure 1) [6,7]. Inactivation of p16 results in a loss of the inhibition of RB phosphorylation, facilitating a loss of control over cell cycle arrest [2]. In the case of breast tumors there may be genetic events upstream of *RB *which can negatively affect RB function by promoting its phosphorylation. These may include p16 loss [5] and *CCND1 *amplification or Cyclin D1 overexpression [8].

CDK4 activity is deregulated in many human tumors [9]. It has been shown that CDK4 and CDK6 are dispensable when it comes to driving the essential cell cycle, but they are required in specialized tissues and possibly for achieving higher levels of proliferation [10,11]. CDK4 has been shown to be absolutely crucial for various oncogenic transformation processes, suggesting that many cancer cells may be addicted to high CDK4 activity [9].

Cyclin D1 is a product of the *CCND1 *gene [12], which is today considered a well-established human oncogene [5]. The gene and its product have been extensively examined in cases of cancer and there is significant evidence of their involvement in breast, lung, colon, bladder and liver cancers, melanoma, oral squamous cell carcinomas and mantle cell lymphoma [4,5,13]. The amplification of *CCND1 *and the overexpression of the cyclin D1 protein frequently occur in breast cancer, although overexpression of the protein is not always due to gene amplification [4,5]. Estrogen uses cyclin D1 as one of its target genes to mediate its mitogenic effects [12]. Cyclin D1 expression has been shown in previous human breast cancer studies to correlate with positive ER status [12,14], the protein being predominantly expressed in the well-differentiated, low-grade, slow-growing subtypes of breast cancer [12].

The product of the *CDKN2 *gene, p16, acts as a tumor suppressor [15], while inactivation of the gene has been found to be a common event in nearly half of all human cancers studied [16]. Normal proliferating cells do not express significant levels of p16 prior to extensive rounds of cell division, which may suggest a late-stage antiproliferative role for p16, as in the senescence of replicative cells [17]. The activation of p16 expression can be triggered by DNA damage, oncogenic stress or physiological aging [7]. The significance of p16 overexpression is not fully understood, however, and it has been associated with both better and poorer prognosis of cancer. p16 has been associated with a poor prognosis for neuroblastoma and cervical, ovarian, breast and prostate tumors [6]. Recent studies have shown a correlation between overexpression of p16 and both an infiltrative tumor border pattern in breast cancer [16] and the basal-like phenotype [18-19].

In the present work, we assessed the expression of cyclin D1, CDK4 and p16 in human invasive ductal mammary carcinoma samples, correlated the findings with the known prognostic factors for breast cancer and investigated the correlations of these three markers with survival functions. As has been said, all the factors studied here contribute to the RB pathway, and the aim was to reveal the significance of cyclin D1 and its regulators, CDK4 and p16, and their interrelations in human breast cancer.

## Methods

### Patient and tumor material

The series consisted of 102 formalin-fixed, paraffin-embedded tumor specimens from female breast cancer patients from the archives of the Department of Pathology, Oulu University Hospital, Finland, dating from the years 2000 to 2007. Informed consents were obtained from the patients. The approval of the local Ethical Committee and the Finnish National Supervisory Authority for Welfare and Health was obtained for the use of the tumor specimens and patient medical records. Information regarding patient characteristics was obtained from the clinical and pathological records. The diagnoses were re-evaluated by the pathologist according to the WHO classification [20] in the course of grading the immunohistochemical stainings. The TNM classification classes T1 to T4 were used to evaluate the tumor size (T1: ≤ 2 cm, T2: > 2 cm but ≤ 5 cm, T3: > 5 cm and T4: tumor of any size, with direct extension to chest wall or skin. The clinical characteristics of the patients are summarized in Table 1.

**Table 1:** Patient characteristics

		*n *= 102	%
Tumor			
	T1	66	64.7
	T2	27	26.5
	T3	6	5.9
	T4	3	2.9
			
Nodus			
	Negative	58	56.9
	Positive	42	41.2
	Unknown	2	1.9
			
Grade			
	I	30	29.4
	II	35	34.3
	III	37	36.3
			
Estrogen receptor (ER)			
	Negative	24	23.5
	Positive	78	76.5
			
Progesterone receptor (PR)			
	Negative	35	34.3
	Positive	67	65.7
			
HER2			
	Negative	84	82.4
	Positive	18	17.6
			
Ki67			
	Negative	21	20.6
	+	37	36.3
	++	20	19.6
	+++	24	23.5
			
Adjuvant treatment			
	Chemotherapy	35	34.3
	Hormonal	44	43.1
	Tamoxifen	23	22.5
	Aromatase		
	inhibitor	17	16.7
	Both	2	2.0
	Unknown	2	2.0
	Radiotherapy	74	72.5
	Trastuzumab	3	2.9

### Immunohistochemistry (IHC) and scoring

#### Tissue processing

The surgical specimens were placed in formalin prior to embedding in paraffin for subsequent routine light microscopy and immunohistochemical analysis of cyclin D1, CDK4 and p16. The immunostaining was carried out as follows. Three micrometer sections were deparaffinized and treated with TRIS/EDTA with pronase for epitope retrieval. After this the sections were incubated with blocking solution (EnVision Detection System, Dako, Glostrup, Denmark) to block nonspecific binding of IgGs and then incubated with polyclonal antibodies to cyclin D1, diluted 1:25 (M3635, Dako North America Inc., Carpinteria, CA, USA), CDK4, diluted 1:100 (DCS-35: sc-23896, Santa Cruz Biotechnology Inc., Santa Cruz, CA, USA), and p16, non-diluted (CINtec, mtm Laboratories AG, Heidelberg, Germany). The color was developed with diaminobenzidine tetrahydrochloride (DAB) (EnVision Detection System, Dako). All the steps were followed by washes with tween/phosphate-buffered saline (PBS). Finally, the sections were lightly counterstained with hematoxylin before analysis by light microscopy and scoring as for DAB staining. For negative controls, the sections were incubated with PBS instead of the primary antibodies. Immunohistochemical analysis of the expression of PHD1-3, HIF-1α and HIF-2α in the same cohort has been described before [21].

#### Scoring

The cytoplasmic and nuclear stainings were evaluated individually for all three markers, and the intensities of both were scored on a scale from 0 to 3, where 0 = negative, 1 = week, 2 = moderate, and 3 = strong staining. Also, the percentage of nuclei was assessed in each case and scored on a scale of 0 to 100%. Evaluation of the staining of PHD1-3, HIF-1α and HIF-2α has been described before [21].

The cut-off points for Ki-67 were: negative < 5%, + 5 to 15%, ++ 16 to 30%, and +++ > 30%. Human Epidermal Growth Factor Receptor 2 (HER2) was considered positive in IHC when the result was either ++ or +++ (reflecting moderate to strong intensity in all or almost all tumor cells), and gene amplification status was determined using chromogenic *in situ *hybridization. Cancers with six or more gene copies were considered HER2 positive [22].

The cut-off value for estrogen and progesterone receptors (ER and PR respectively) was 10%. Tumor samples with ER or PR expression less than 10% were considered negative and the others positive.

### RNA extraction, cDNA synthesis and quantitative real time PCR (Q-PCR)

RNA was extracted from the paraffin sections of breast tumors with NucleoSpin FFPE RNA/DNA (Macherey-Nagel, Düren, Germany). A total of 100 ng of RNA was used for cDNA synthesis performed with iScript (Bio-Rad, Hercules, CA, USA). The amount of cyclin D1 mRNA relative to 18S rRNA in the samples was analyzed by Q-PCR performed in a Stratagene MX3005 thermocycler with iTaq SYBR Green Supermix and ROX (Bio-Rad) and the primers qHs3CCND1For 5'-GCTCCTGGTGAACAAGCTCAA-3', qHs3CCND1Rev 5'-TTGGAGAGGAAGTGTTCAATGAAA-3', Hs18SFor 5'-GACTCAACACGGGAAACCTC-3' and Hs18SRev 5'-AGCATGCCAGAGTCTCGTTC-3', respectively.

### Statistical analyses

The statistical analyses were carried out with SPSS 17.0 (SPSS Inc., Chicago IL, USA). The clinical characteristics were expressed as percentages. The Chi-square test or Fisher's exact test was used to evaluate the associations and correlations, as appropriate. A two-tailed *P*-value was used in all the analyses, and a *P*-value < 0.05 was considered statistically significant. Disease-free survival, breast cancer-specific survival and overall survival were analyzed by the Kaplan-Meier method using Cox regression. Hazard ratios (HR) and 95.0% confidence intervals (95.0% CI) are indicated. Disease free survival was considered as the primary endpoint.

For the statistical analyses, the staining results for the proteins studied were combined to form a positive and a negative group. Cyclin D1 was considered positive when the proportion of stained nuclei was > 40% and when the intensity of the nuclear staining was strong and negative in other cases. CDK4 and p16 were scored as negative when ≤ 2% of the nuclei were stained and the nuclear intensity was negative or weak and as positive in other cases.

## Results

All the 102 patients were women and their median age was 59 years (range 28 to 87 years). The prognostic factors are described in Table 1. The majority of the tumors were of low tumor stage (64.7% were T1), nodal negative (56.9%), and steroid receptor positive (76.5% were ER positive and 65.7% PR positive). A total of 17.6% of the tumors were HER2 positive. Grades I to III were represented in almost equal numbers. About 40% of the cancers were intermediate or highly proliferated. The adjuvant treatments provided for the patients are described in Table 1. Only 12% of the patients did not receive any adjuvant therapy (Table 1).

The purpose here was to correlate the immunohistochemical expression of cyclin D1, CDK4 and p16 with the main clinical prognostic factors for breast cancer: tumor stage, nodal status, tumor grade, steroid receptors (ER and PR), HER2 status and proliferation rate.

**Figure 2:**
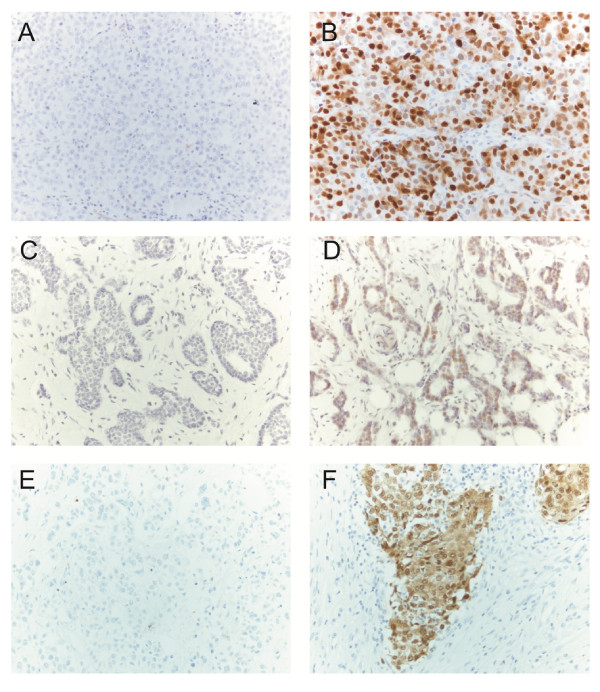
**Cyclin D1, CDK4 and p16 expression in invasive ductal breast carcinomas analyzed by immunohistochemistry **. **(B) **High nuclear cyclin D1 expression. **(D) **High nuclear CDK4 expression. **(F) **High p16 expression. **(A)**, **(C) **and **(E) **are negative controls for (B), (D) and (F), respectively.

Cyclin D1 showed nuclear and cytoplasmic staining (Figure 2A, B), and CDK4 only nuclear staining (Figure 2C, D), while p16 staining was mainly detected in the nuclei and to a lesser extent in the cytoplasm (Figure 2E, F). It is also significant that the presence of lymphocyte nuclei always meant positivity for cyclin D1 and that of fibroblasts often for p16 (data not shown).

**Figure 3:**
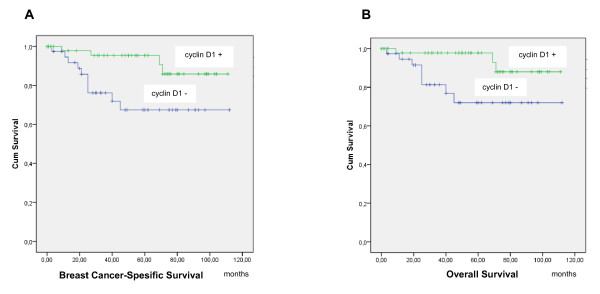
**Kaplan-Meier curves for breast cancer-specific and overall survival stratified by cyclin D1 expression**. **(A) **Breast cancer-specific survival of patients with high tumor cyclin D1 expression relative to negative or low expression (*P *= 0.020). **(B) **Overall survival of patients with high tumor cyclin D1 expression relative to negative or low expression (*P *= 0.013).

About 60% of the stained specimens showed nuclear cyclin D1 expression. Statistically significant correlations emerged between cyclin D1 and a lower tumor grade (*P *= 0.013), ER and PR positivity (*P *= 0.000, *P *= 0.024, respectively) and a negative or low proliferation rate (*P *= 0.031) (Table 2). Likewise, cyclin D1 expression was shown to correlate with increased breast cancer-specific survival (*P *= 0.020; HR = 4.26; 95.0% CI = 1.12, 16.1) (Figure 3A) and increased overall survival (*P *= 0.013; HR = 3.93; 95.0% CI = 1.23, 12.6) (Figure 3B), but it had no significant association with disease-free survival (data not shown).

**Table 2:** Cyclin D1, CDK4 and p16 expression in breast cancer in relation to clinicopathological variables

	Cyclin D1 - n (%)	Cyclin D1 + n (%)	*P-*value	Cdk4 - n (%)	Cdk4 + n (%)	*P-*value	P16 - n (%)	p16 + n (%)	*P-*value
Tumour									
T1	21 (52.5)	41 (73.2)		14 (56.0)	39 (65.0)		27 (64.3)	32 (62.7)	
T2	13 (32.5)	12 (21.4)		10 (40.0)	14 (23.4)		10 (23.8)	15 (29.4)	
T3	3 (7.5)	3 (5.4)		1 (4.0)	5 (8.3)		2 (4.8)	4 (7.8)	
T4	3 (7.5)	0 (0.0)		0 (0.0)	2 (3.3)		3 (7.1)	0 (0.0)	
			0.066			0.431			0.273
Nodus									
Negative	20 (51.3)	34 (61.8)		15 (65.2)	34 (56.7)		26 (61.9)	29 (59.2)	
Positive	19 (48.7)	21 (38.2)		8 (34.8)	26 (43.3)		16 (38.1)	20 (40.8)	
			0.309			0.478			0.791
Grade									
I	9 (22.5)	20 (35.7)		11 (44.0)	14 (23.3)		11 (26.2)	17 (33.3)	
II	10 (25.0)	23 (41.1)		6 (24.0)	22 (36.7)		14 (33.3)	16 (31.4)	
III	21 (52.5)	13 (23.2)		8 (32.0)	24 (40.0)		17 (42.5)	18(35.3)	
			0.013*			0.156			0.747
Estrogen receptor (ER)									
Negative	18 (45.0)	3 (5.4)		6 (24.0)	14 (23.3)		10 (23.8)	12 (23.5)	
Positive	22 (55.0)	53 (96.6)		19 (76.0)	46 (76.7)		32 (76.2)	39 (76,5)	
			0.000***			0.947			0.975
Progesterone receptor (PR)									
Negative	18 (45.0)	13 (23.3)		8 (32.0)	22 (36.7)		15 (35.7)	18 (35.3)	
Positive	22 (55.00)	43 (76.8)		17 (68.0)	38 (63.3)		27 (64.3)	33 (64.7)	
			0.024*			0.682			0.966
HER2									
Negative	30 (75.0)	48 (85.7)		21 (84.0)	48 (80.0)		28 (66.7)	47 (92.2)	
Positive	10 (25.0)	8 (14.3)		4 (16.0)	12 (20.0)		14 (33.3)	4 (7.8)	
			0.185			0.769			0.002**
Ki67									
Negative	8 (20.0)	12 (21.4)		6 (24.0)	9 (15.0)		8 (19.0)	10 (19.6)	
+	10 (25.0)	24 (42.9)		11 (44.0)	21 (35.0)		15 (35.7)	18 (35.3)	
++	7 (17.5)	13 (23.2)		2 (8.0)	16 (26.7)		10 (23.8)	9 (17.6)	
+++	15 (37.5)	7 (12.5)		6 (24.0)	14 (23.3)		9 (21.4)	14 (27.5)	
			0.031*			0.251			0.856

* *P *< 0.05, ** *P *< 0.01, *** *P *< 0.001.

**Figure 4:**
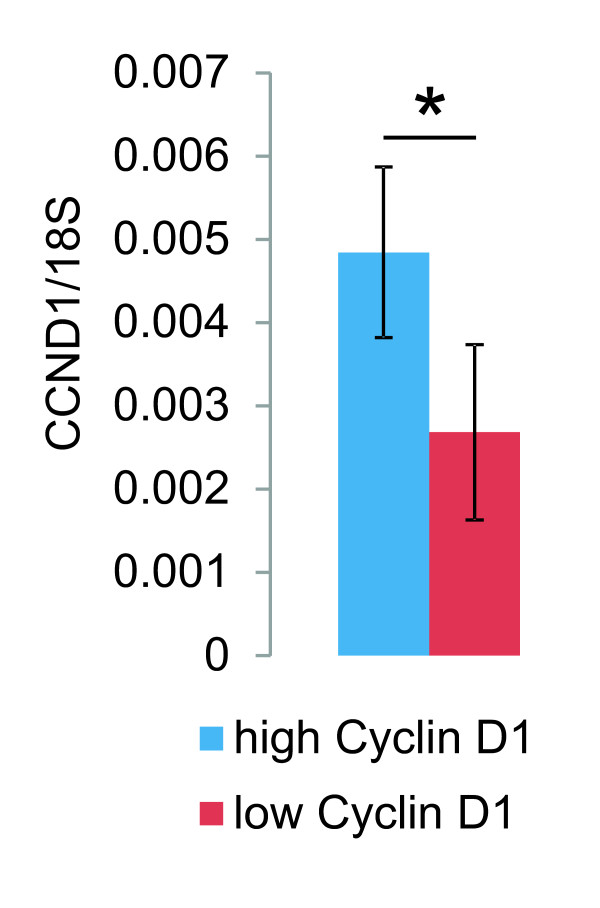
**Relative expression of cyclin D1 mRNA to cyclin D1 protein in breast carcinoma specimens**. Expression of cyclin D1 mRNA was studied relative to 18S rRNA (CCND1/18S). High and low cyclin D1 indicate the expression levels of the protein.

Evaluation of the relation of cyclin D1 protein expression in the histological sections to cyclin D1 mRNA in the corresponding tumor samples by means of Q-PCR showed that tumors with high cyclin D1 protein had 1.8 times higher expression of cyclin D1 mRNA than those with low or negative cyclin D1 in the histological sections (Figure 4), suggesting that the higher cyclin D1 protein level is at least partially due to increased expression of *CCND1*.

Although about 70% of the tumor samples showed nuclear CDK4 expression, no statistically significant correlation was found between this and any of the clinico-pathological factors studied (Table 2). There was, however, a tendency for CDK4 expression to correlate with a high tumor grade (*P *= 0.156) (Table 2), but not with any of the survival functions (data not shown).

**Figure 5:**
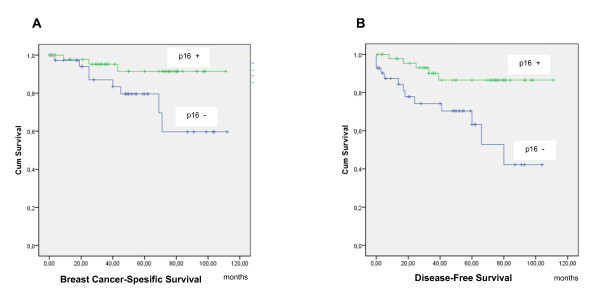
**Kaplan-Meier curves for breast cancer-specific and disease-free survival stratified by p16 expression**. **(A) **Breast cancer-specific survival of patients with high tumor p16 expression relative to negative or low expression (*P *= 0.028). **(B) **Disease-free survival of patients with high tumor p16 expression relative to negative or low expression (*P *= 0.004).

Nuclear p16 expression was seen in 55% of the breast carcinoma specimens. It correlated significantly only with HER2 negativity (*P *= 0.022). In addition, we found a statistically significant correlation between p16 expression and increased breast cancer-specific survival (*P *= 0.028; HR = 4.0; 95.0% CI = 1.0, 15.0) (Figure 5A) and increased disease-free survival (*P *= 0.004; HR = 4.1; 95.0% CI = 1.5, 12.0) (Figure 5B), but not with overall survival (data not shown).

The interrelations between the above three markers were analyzed, as they are linked together in the cell cycle, but no statistically significant correlation was found between cyclin D1 and CDK4 or between CDK4 and p16. There was a trend for a positive correlation between cyclin D1 and p16, however (*P *= 0.145). No significant correlation among all three factors was observed in a three-variable logistic regression model.

When we considered the relations between the expression of cyclin D1, CDK4 and p16 and hypoxia-inducible factor (HIF) prolyl 4-hydroxylases PHD1, 2 and 3, HIF-1α and HIF-2α, the expression of which we had studied earlier in the same samples [21], we found positive correlations between p16 and both PHD1 (*P *= 0.032) and PHD2 (*P *= 0.027) and a correlation between CDK4 positivity and PHD3 negativity (*P *= 0.031).

## Discussion

Disruption of the p16-cyclin D1-CDK4/6-RB pathway occurs frequently in many human cancers [2], and we investigated here the expression profiles of three distinct factors involved in this pathway: cyclin D1, CKD4 and p16. Overexpression of cyclin D1 has earlier been associated with breast cancer subtypes that are more indolent, estrogen receptor positive, and have a better prognosis [12], and our findings pointed to a similar pattern. Cyclin D1 was statistically significantly correlated with estrogen and progesterone receptor positivity, a lower tumor grade and lower proliferation activity, that is, with breast cancers that have a good prognosis. In addition, high cyclin D1 expression significantly increased breast cancer-specific survival and overall survival in our cohort. Gene amplification of *CCND1 *has been reported to be associated with an increased risk of breast cancer recurrence, while nuclear expression of cyclin D1 protein was associated with a decreased recurrence rate [23]. Amplification of the *CCND1 *gene has been identified in approximately 15 to 20% of human breast cancers, while overexpression of cyclin D1 protein has been demonstrated in 50 to 70% [5]. Our data showed a correlation between a high cyclin D1 mRNA level and high cyclin D1 protein expression. Hence, at least some of the increased protein expression of cyclin D1, if not all, must be due to the amplification of the corresponding gene. The prognostic significance of cyclin D1 overexpression with respect to cancer in general does not seem to be consistent, however. High expression of cyclin D1 has been associated with resistance to tamoxifen therapy [24-26]. And in another hormone-dependent cancer type, prostate cancer, overexpression of cyclin D1 is associated with a high proliferative index and a metastatic disease, and it has been suggested that high cyclin D1 may be related to the evolution of an androgen-independent disease form [27].

Although CDK4 was expressed in about 70% of our tumor samples, it surprisingly did not show any significant correlations with the main clinical prognostic factors for breast cancer nor with the survival functions. A tendency for CKD4 positivity to correlate with a higher tumor grade was noticed, however, coinciding with the notion that CDK4 promotes tumor cell proliferation [10]. This is not entirely surprising since others have reported earlier in a similar size German cohort that even though 16% of sporadic breast cancers overexpressed CDK4, no association between any of other clinical factors, except Ki-67, was observed [28]. It is thought that the ability of cyclin D1 to activate CDK4 is critical for driving tumorigenesis, and that CDK4-associated kinase activity is required to maintain this tumorigenesis, as shown in mice with Her2-induced, but not Wnt-1-induced [29] and Ras-induced breast cancer [30]. However, we did not find any correlation between CDK4 and cyclin D1 expression. It has, however, been reported that the oncogenic activity of cyclin D1 in human cancers is independent of CDKs [31]. Not only does the interaction between CDK4/6 and cyclin D1 suggest that they act interdependently, but cyclin D1 has also been reported to function independently of CDK4/6 in supporting proliferation by directly activating estrogen receptors [12]. As noted previously, our data point to a high correlation between positive ER status and cyclin D1 expression, which supports the suggestion of a direct estrogen receptor-mediated mode of function for cyclin D1. Collectively, these data together with some previous data [28] suggest that the role of CDK4 in human breast carcinogenesis differs from that in mouse and further emphasizes the importance of clinical studies.

As a tumor suppressor, p16 is a negative cell cycle regulator, and its inactivation appears to be a common event in many cancers [16], and in many cases it is associated with poorer prognosis. For example, p16 overexpression has been found in high-grade carcinomas of the oropharynx and the genital and genitourinary tracts [32-34]. Yet the role of its overexpression in human breast cancer is a point of controversy. We found a significant correlation between high p16 expression and HER2 negativity, and expression of the oncogene *HER2 *is considered a very poor prognostic factor for breast cancer [35]. An association of ER negativity, higher grading and high proliferation activity with the overexpression of 16 has been detected in previous breast cancer studies [36], as also has its association with the basal-like phenotype [18,19]. However, in our cohort, statistically significant correlations between high p16 expression and both increased disease-free survival and increased breast cancer-specific survival were observed.

The expression of p16 has been shown to correlate with the inhibition of VEGF and angiogenesis, but the mechanism by which p16 regulates VEGF has not been properly explored. One hypothesis is that the activity of HIF-1α, which is responsible for hypoxia-induced malignant progression, including VEGF transactivation, can be attenuated by p16 [37]. Moreover, p16 itself has been shown to be hypoxia-inducible [38]. On the other hand, we did not find any correlation between p16 and HIF-1α or HIF-2α, having studied the expression of both of these in the same cohort earlier [21]. The HIF prolyl 4-hydroxylases, PHD1-3, are oxygen sensors that negatively regulate HIF-α [39]. In the present work, p16 had a positive correlation with PHD1 and PHD2, while CDK4 had an inverse correlation with PHD3. The levels of expression of PHD1-3 have been determined in the same set of samples earlier [21], and similarly to high p16 expression, high PHD2 expression had a tendency to promote breast cancer-specific survival and disease-free survival [21]. The most significant correlation found earlier for PHD3 was with a low tumor grade (*P *= 0.000) [21], while interestingly, there was a tendency for high CDK4 expression to correlate with a high tumor grade, supporting the identified inverse relation between these factors. PHD3 depletion under hypoxia has been shown in a recent study involving a head and neck squamous cell carcinoma line to be associated with cell cycle arrest at the G1/S interface, in that it reduces the amount of hyperphosphorylated RB [40].

To our knowledge, this is the first time when the interrelation between cyclin D1, CDK4 and p16 has been studied in human breast cancer patient samples. We show in this cohort that the expression of two of the studied markers, p16 and cyclin D1, correlates with better prognosis of breast cancer. A significant association of these factors in the rather small cohort with limited number of events highlights the strength of these findings. Our data on cyclin D1 and CDK4 agree with those in some earlier patient studies [12,14,28]. The failure to identify an interrelation among cyclin D1, CDK4 and p16 may be due to a limited cohort size, or a more complex interrelation beyond the protein level expressions studied here.

## Conclusions

In summary, high p16 expression correlated with HER2 negativity and longer breast cancer-specific and disease-free survival in human breast cancer. In addition, our data confirm earlier findings correlating high cyclin D1 expression with a good prognosis for breast cancer. The expression levels of cyclin D1 protein and mRNA correlated with each other. Surprisingly, no significant correlations among cyclin D1, CDK4 and p16 were found in terms of their expression.

## Abbreviations

CDK: cyclin-dependent kinase; CI: confidence interval; DAB: diaminobenzidine tetrahydrochloride; ER: estrogen receptor; HIF: hypoxia inducible factor; HR: hazard ratio; IHC: Immunohistochemistry; PBS: phosphate-buffered saline; PR: progesterone receptor; Q-PCR: quantitative real time PCR; RB: retinoblastoma.

## Competing interests

The authors declare that they have no competing interests.

## Authors' contributions

EP, PK, KMH and AJV participated in the study design. EP was also involved in data collection, data interpretation and the statistical analyses and the writing of the manuscript. PK, KMH and AJV contributed equally to the data collection, analysis and interpretation, and to the writing of the manuscript. RB performed the statistical analyses. All authors read and approved the final manuscript.
